# Extreme Thrombocytosis in Patients with Overt Myelofibrosis and Its Clinical Associations

**DOI:** 10.3390/cancers17091390

**Published:** 2025-04-22

**Authors:** Marko Lucijanic, Ivan Krecak, Ena Soric, Anica Sabljic, Davor Galusic, Hrvoje Holik, Vlatka Perisa, Martina Moric Peric, Ivan Zekanovic, Leonardo Budimir, Rajko Kusec

**Affiliations:** 1 Hematology Department, University Hospital Dubrava, Av. Gojka Suska 6, 10000 Zagreb, Croatia; 2 Scientific Research and Translational Medicine Department, University Hospital Dubrava, Av. Gojka Suska 6, 10000 Zagreb, Croatia; 3 School of Medicine, University of Zagreb, Ul. Salata 3, 10000 Zagreb, Croatia; 4 Department of Internal Medicine, General Hospital Sibenik, Ul. Stjepana Radica 83, 22000 Sibenik, Croatia; 5 School of Medicine, University of Rijeka, Ul. Brace Branchetta 20/1, 51000 Rijeka, Croatia; 6 Sibenik University of Applied Science, Trg Andrije Hebranga 11, 22000 Sibenik, Croatia; 7 Department of Hematology, University Hospital of Split, Soltanska ul. 1, 21000 Split, Croatia; 8 School of Medicine, University of Split, Soltanska ul. 2, 21000 Split, Croatia; 9 Department of Internal Medicine, “Dr. Josip Bencevic” General Hospital, Ul. Andrije Stampara, 35000 Slavonski Brod, Croatia; 10 Faculty of Medicine, University of Osijek, Ul. Josipa Huttlera 4, 31000 Osijek, Croatia; 11 Department of Hematology, Osijek University Hospital, Ul. Josipa Huttlera 4, 31000 Osijek, Croatia; 12 Department of Internal Medicine, General Hospital Zadar, Ul. Boze Pericica 5, 23000 Zadar, Croatia

**Keywords:** chronic myeloproliferative neoplasm, BCR::ABL negative, primary myelofibrosis, polycythemia vera, essential thrombocythemia

## Abstract

**Background/Objectives**: Overt primary myelofibrosis (PMF), secondary post-polycythemia vera (post-PV), and post-essential thrombocythemia (post-ET) myelofibrosis (SMF) are chronic myeloproliferative neoplasms (MPN) that sometimes present with extreme thrombocytosis (ExTh, platelet count > 1000 × 10^9^/L), a phenomenon of uncertain clinical significance since there are no published data available. **Methods**: We retrospectively investigated the clinical correlations and associated outcomes of ExTh in a cohort of 172 patients with overt myelofibrosis diagnosed in six Croatian hematology centers. **Results**: ExTh was present in 5.8% of patients and was associated with post-ET etiology of myelofibrosis, older age, smaller spleen size, and the presence of arterial hypertension (*p* < 0.05 for all analyses). No significant associations were observed with sex, degree of bone marrow fibrosis, or driver mutation status. Over the follow-up period, patients with ExTh experienced a favorable course regarding survival (*p* < 0.001) and bleeding risk (*p* = 0.034), whereas no significant association with thrombotic risk was observed (*p* = 0.682). **Conclusions**: In contrast to its context in ET, ExTh in overt fibrotic MPN does not appear to confer higher bleeding or thrombotic risk. Instead, it is associated with more favorable survival outcomes and reduced bleeding risk.

## Simple Summary

Extreme thrombocytosis (ExTh, platelet count > 1000 × 10^9^/L) is observed in some patients diagnosed with primary or secondary myelofibrosis, a specific tumor of the hematopoietic stem cell. Nevertheless, there are no data in the literature on this phenomenon that may aid in clinical decision-making when it is present. Here, we report the incidence, clinical correlations, and associated outcomes of ExTh. As our study shows, ExTh is rare but may represent a favorable phenomenon.

## 1. Introduction

Chronic myeloproliferative neoplasms (MPNs) are malignant disorders of the hematopoietic stem cells, resulting in their uncontrolled proliferation and the production of mature-appearing blood cells [1,2]. Stem cell proliferation is driven by upregulation of the Janus kinase (JAK)–signal transducer and activator of transcription (STAT) signaling pathway, as a consequence of acquired mutations in JAK2, calreticulin (CALR), or thrombopoietin receptor (myeloproliferative leukemia proto-oncogene, MPL) genes in the majority of patients [3]. This, in turn, drives not only proliferation but also the strong inflammatory milieu characteristic of MPNs [4]. There are three classical entities: polycythemia vera (PV), essential thrombocythemia (ET), and primary myelofibrosis (PMF). PMF can present as pre-fibrotic disease or as overt fibrotic disease with developed clinical complications, including a high grade of bone marrow fibrosis, splenomegaly, anemia, and the presence of constitutional symptoms [5,6]. PV and ET may also develop overt bone marrow fibrosis, which is accompanied by the development of similar clinical complications—a state termed secondary myelofibrosis (SMF) [7]. The clinical presentation and prognosis of overt fibrotic PMF and SMF overlap, and both diseases respond to the same therapies, although some differences exist at the molecular and clinical levels [8,9].

A high thrombotic risk is inherent to all MPN subsets [1,10]. MPN patients tend to develop both arterial and venous thromboses, and clinical risk scores that guide the introduction of cytoreductive therapies are based on the prediction of thrombotic risk. An increased risk of bleeding is also characteristic of MPN patients; however, it is often a secondary consideration in clinical decision-making [11]. When patients progress to the overt myelofibrosis stage, the focus of prognostication and treatment shifts to the evaluation and reduction of mortality risk. Nevertheless, thrombotic risk remains high and is usually insufficiently managed [12].

Complete blood count and its components are the mainstay of evaluation for MPN patients [13]. Although MPN patients in prefibrotic stages typically present with cytoses, they tend to develop anemia, thrombocytopenia, and in some instances, leukopenia when progressing to the overt fibrotic stage [14]. Among patients with developed myelofibrosis, so-called myelodepletive and cytopenic phenotypes are associated with poor prognosis and a higher risk of death [15]. Thrombocytopenia is a relevant clinical issue and is recognized as a negative prognostic parameter in patients with myelofibrosis, incorporated into prognostic scores [16]. It limits the introduction and dosing of myelofibrosis-specific therapies, as well as antiplatelet and anticoagulant medications. On the other side of the platelet spectrum, a proportion of patients present with elevated platelet counts (>450 × 10^9^/L). These patients usually tolerate cytoreductive therapies better and are more reminiscent of prefibrotic MPN patients [17].

Extreme thrombocytosis (ExTh) is a presenting feature in some MPN patients, usually associated with the ET or prefibrotic PMF phenotype [18]. It is typically defined as a platelet count greater than 1500 or 1000 × 10^9^/L, with the cut-off value being more psychological than clinical. However, among ET patients, those with ExTh tend to have a higher bleeding risk linked to aspirin use [19], which is in part due to acquired von Willebrand syndrome [20,21], and ExTh is considered a valid indication for the initiation of cytoreductive therapies regardless of other clinical features and risks [22]. ExTh can be encountered in all MPN contexts, even among overt fibrotic MPN patients. However, there are no data in the literature on its clinical significance in the context of overt myelofibrosis. Since the clinical significance of this phenomenon is uncertain, we aimed to investigate the clinical correlations of ExTh (defined as a platelet count > 1000 × 10^9^/L) at the time of diagnosis among overt-stage PMF and SMF patients, and the associated clinical risks.

## 2. Materials and Methods

### 2.1. Patients and the Methodology

We conducted a retrospective analysis of a multicenter cohort of patients with overt myelofibrosis. The study included 172 patients who were diagnosed and treated in six Croatian hematology centers between 2004 and 2025. All diagnoses were reassessed according to the World Health Organization (WHO) 2022 [23] and the International consensus classification (ICC) 2022 criteria [24]. Bone marrow biopsy was mandatory for inclusion, and the degree of bone marrow fibrosis was graded according to the European Consensus criteria [25]. Disease severity was categorized using either the Dynamic International Prognostic Scoring System (DIPSS) [26] in PMF patients or the Myelofibrosis Secondary to PV and ET Prognostic Model (MYSEC-PM) in SMF patients [27]. Comorbidities were assessed both as individual entities and as a cumulative comorbidity burden, measured by the Charlson Comorbidity Index [28]. Platelet count, in addition to other laboratory and clinical parameters, was assessed at baseline, and the results are presented from the perspective of ExTh, defined as platelets > 1000 × 10^9^/L [21]. Platelet count was further stratified into <150, 150–450, and 450–1000 × 10^9^/L categories.

The study was approved by the Institutional Review Boards of the University Hospital Dubrava (2020/0306-05; 8 June 2020), the University Hospital Center Split (2181-147-01/06/M.S.-19-3; 31 January 2019), the University Hospital Center Osijek (R2-1060/2020; 25 February 2020), the General Hospital Zadar (02-2025/20-6/20; 10 April 2020), the General Hospital of Sibenik-Knin County (01-3618/1-20; 26 February 2020) and the Dr. Josip Bencevic General Hospital (04000000/20-37; 20 May 2020). All procedures were in accordance with the ethical standards of the responsible committee on human experimentation (institutional and national) and with the Helsinki Declaration of 1975, as revised in 2008.

### 2.2. Statistical Methods

The normality of the distribution of numerical variables was tested using the Shapiro–Wilk test. Due to the non-normal distribution of the majority of variables, all numerical variables were presented as medians with interquartile ranges (IQR) and analyzed using non-parametric statistical tests. Comparisons of numerical variables between groups were performed using the Mann–Whitney U test. Categorical variables were expressed as ratios or percentages and compared between groups using the Chi-square or Fisher’s exact test, as appropriate.

Time-to-event analyses were performed using the Kaplan–Meier method. Patients were evaluated from diagnosis until death or their last known visit for overall survival (OS), from diagnosis to the first arterial or venous thrombotic event for time to thrombosis (TTT), and from diagnosis to the first bleeding event for time to bleeding (TTB). Arterial thrombotic events considered included myocardial infarction, cerebrovascular infarction, and peripheral arterial thrombosis. Venous thrombotic events considered included deep venous thrombosis, pulmonary embolism, and cerebral or splanchnic venous thrombosis. The Cox–Mantel version of the log-rank test was used to compare time-to-event data between groups [29]. Multivariate time-to-event analyses were conducted using Cox regression analysis [30]. Screening of time-to-event associations was performed using a custom-developed Microsoft Excel workbook [31].

*p* values < 0.05 were considered statistically significant. All presented analyses were performed using the MedCalc statistical program version 23.0.2 (MedCalc Software Ltd., Ostend, Belgium).

## 3. Results

**Figure 1:**
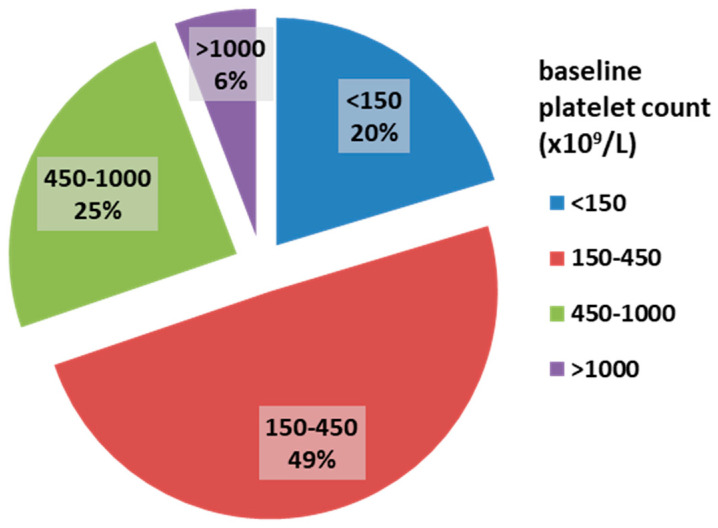
Frequencies of platelet count categories in patients with overt myelofibrosis at baseline.

Among the 172 myelofibrosis patients, 58.7% were male. The median age was 68 years (IQR 62–76). JAK2 V617F, CALR, and MPL mutations were present in 74.1%, 8.1%, and 3% of patients, respectively. The majority of patients had grade II bone marrow fibrosis (112 patients, 65.1%), while 60 patients (34.9%) had grade III fibrosis. The median platelet count was 316 × 10^9^/L. ExTh was present in 10 patients (5.8%); platelet counts in the range of 450–1000 × 10^9^/L were observed in 42 patients (24.4%), normal platelet counts (150–450 × 10^9^/L) in 85 patients (49.4%), and thrombocytopenia in 35 patients (20.4%). The distribution of patients based on baseline platelet count is presented in Figure 1.

The relationship between patients’ characteristics and the presence of ExTh is presented in Table 1. Patients with post-ET SMF were the most likely to have ExTh (14.7%), followed by those with PMF (5%) and post-PV SMF (0%) (*p* = 0.025). ExTh at the time of MF diagnosis was significantly associated with older age (*p* = 0.009), smaller palpable spleen size (*p* = 0.015), and a higher likelihood of cardiovascular risk factors (*p* = 0.034), namely arterial hypertension. ExTh was also associated with higher serum creatinine concentration (*p* = 0.007) and lower MCHC (*p* = 0.013). No significant associations were found with respect to sex, leukocyte and erythrocyte counts, JAK2, CALR, or MPL mutational status, or stage of bone marrow fibrosis. Additionally, no significant association was observed with the DIPSS or MYSEC-PM risk stratification systems (*p* > 0.05 for all analyses). Patients with ExTh were more likely to receive aspirin (*p* = 0.015), whereas no differences were observed regarding other specific therapies.

All patients with ExTh received cytoreductive therapy aimed at reducing platelet count. The majority of patients received hydroxyurea (nine patients), and one patient received interferon. One patient experienced early death due to a thrombotic event (pulmonary embolism), whereas a trend of reduction in platelet count at 6 months was observed in all remaining patients, with a median platelet count of 530 × 10^9^/L at 6 months (median 43% of baseline platelet count).

**Figure 2:**
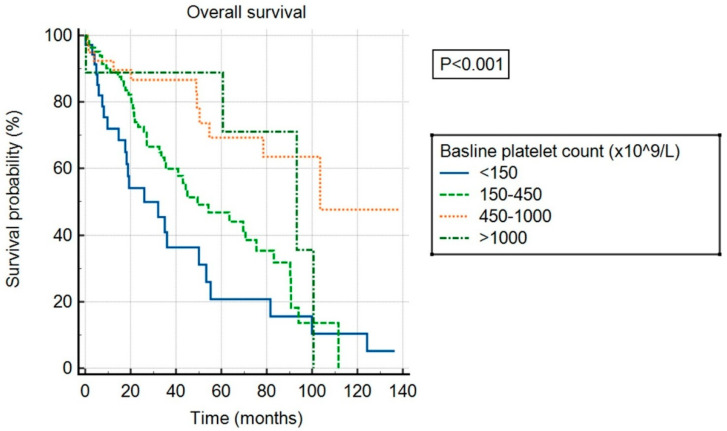
Overall survival of patients stratified by baseline platelet count.

Over the follow-up period, a total of 84 patients died, 23 experienced a thrombotic event, and 13 experienced a bleeding event. The median follow-up was 56 months. Median OS was 55 months in the overall cohort and differed significantly between patients in different baseline platelet count categories (*p* < 0.001), as shown in Figure 2. Patients with ExTh experienced a favorable course regarding OS, comparable to patients with platelet counts of 450–1000 × 10^9^/L, with 36-month survival rates of 88.9% and 86.7%, respectively, whereas survival rates deteriorated with lower baseline platelet counts, being 60% and 36.4% at 36 months in patients with normal and low baseline platelet counts, respectively. Having platelets above normal at baseline persisted as an independent predictor of higher OS in a multivariate model adjusted for DIPSS covariates, independently of other predictors: high platelets (HR 0.43, 95% CI [0.27–0.69], *p* < 0.001), age > 65 years (HR 2.55, 95% CI [1.65–3.94], *p* < 0.001), WBC > 25 (HR 2.59, 95% CI [1.55–4.32], *p* < 0.001), circulating blasts ≥ 1% (HR 1.68, 95% CI [1.1–2.57], *p* = 0.015), hemoglobin < 100 g/L (HR 2.14, 95% CI [1.42–3.2], *p* < 0.001), and presence of constitutional symptoms (HR 1.74, 95% CI [1.13–2.67], *p* = 0.016).

**Figure 3:**
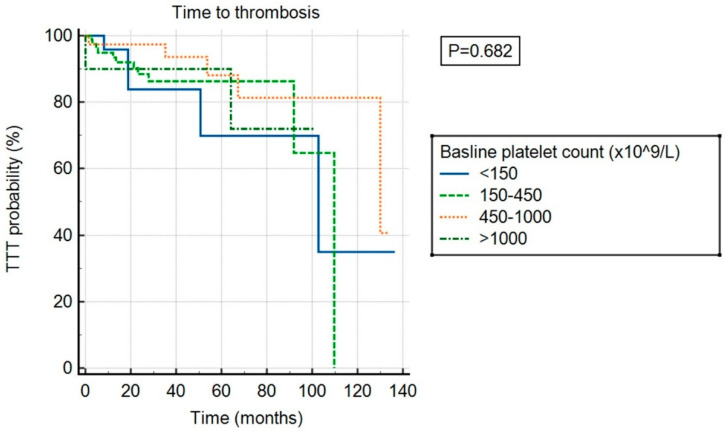
Time to thrombosis (TTT) stratified by baseline platelet count.

**Figure 4:**
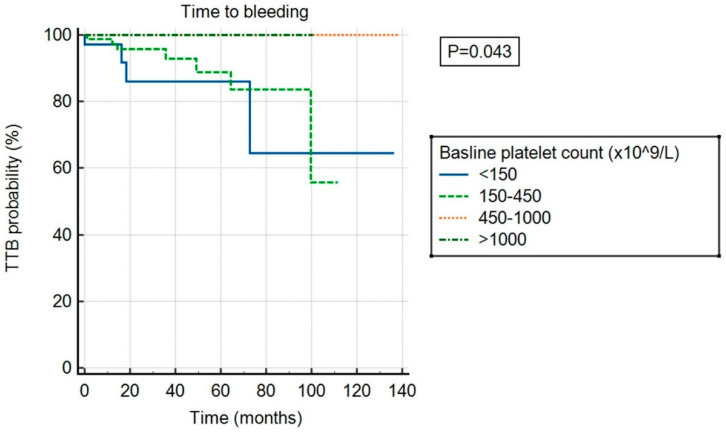
Time to bleeding (TTB) stratified by baseline platelet count.

There was no significant association between ExTh and thrombotic risk (*p* = 0.682) compared to other platelet count categories, as shown in Figure 3. However, ExTh patients, similar to those with platelet counts of 450–1000 × 10^9^/L, had a favorable bleeding risk over time compared to patients with normal or low platelet counts (*p* = 0.043), as shown in Figure 4.

## 4. Discussion

To the best of our knowledge, this study is the first to evaluate the phenomenon of ExTh in patients with overt myelofibrosis, its clinical correlations, and associated risks. As our data suggest, the presence of ExTh is rare but more prevalent among patients with post-ET SMF (14% of patients in this subgroup) and may signal the progression of ET into the myelofibrotic stage. In contrast, PV patients do not appear to develop ExTh at the time of progression, or it is sufficiently rare that it was not encountered in our cohort of patients.

Dysregulation in megakaryopoiesis is one of the key processes in the biology of MPN, regardless of phenotype or peripheral platelet count [32,33]. In addition to being part of the malignant clone, megakaryocytes further contribute to the tumor microenvironment through the production of cytokines such as transforming growth factor beta (TGF-β), which may promote the development of fibrosis and drive progression of the stem-cell disease into more aggressive malignant forms [34,35,36]. The strength of platelet production becomes very important when patients develop overt myelofibrosis [37] and a significant proportion of patients present with low platelet counts [38]. This occurs due to the replacement of hematopoietic bone marrow tissue with fibrosis and the inability of extramedullary hematopoiesis to compensate effectively by producing adequate platelet counts [39,40]. Additionally, massive splenomegaly leads to platelet sequestration, further contributing to thrombocytopenia [41]. Patients who present with a low platelet count tend to have high-risk karyotypes and genetic abnormalities associated with poor prognosis (such as SRSF2 and TP53 mutations) [38,42,43]. Besides MPNs, bone marrow fibrosis can also be encountered in the context of other hematologic and solid malignancies, such as myelodysplastic syndrome (MDS) [44], hairy cell leukemia [45], metastatic cancer [46], and others, and is usually associated with a lower platelet count and poor prognosis [47,48]. Thrombocytopenia in myelofibrosis patients is recognized as a feature with a negative prognosis in the DIPSS-plus prognostic score [16], and because it is associated with difficulty in achieving optimal dosing of standard therapies, it highlights the need to consider more aggressive treatment approaches, such as allogeneic hematopoietic stem cell transplantation, in a timely manner. Conversely, poor-risk features are less frequent among patients with higher platelet counts. Higher platelet counts allow for better tolerance of JAK inhibitors [37] and the introduction of full doses of both antiplatelet and anticoagulant therapies if needed [49], especially for the treatment of cardiovascular comorbidities, which are frequent in MPN patients [50].

ExTh is a known and clinically relevant problem among ET patients. It is associated with a higher bleeding risk and requires the attention of treating physicians [51]. The underlying mechanism of the paradoxically higher bleeding risk associated with elevated platelet counts has been identified as the acquired loss of high-molecular-weight von Willebrand factor multimers, leading to acquired von Willebrand syndrome [51,52]. Cytoreduction is often considered in this context, although the risks and benefits are not completely clear [51,53,54]. Reactive thrombocytosis without underlying MPN also occurs in clinical practice and, in contrast, appears to be associated with a higher thrombotic risk; however, no recommendations exist for specific treatment besides controlling the primary process driving the inflammation [55]. Myelofibrosis patients have a high bleeding risk due to the frequent development of bleeding-prone sites, liver dysfunction from extramedullary hematopoiesis, sometimes low platelet counts, and exposure to therapies from earlier MPN stages that are designed to reduce thrombotic risk (e.g., aspirin) [56,57]. As our data show, myelofibrosis patients with ExTh were more frequently treated with aspirin than those without it, probably in part due to the association with a higher proportion of cardiovascular comorbidities, mostly arterial hypertension. It should also be noted that at the time of SMF diagnosis, a high proportion of patients had already received cytoreductive therapy, and ExTh developed despite such treatment. However, ExTh had no significant association with prior or other specific therapies utilized for myelofibrosis treatment, which is most likely due to low statistical power resulting from the small number of patients in specific subgroups.

The presence of ExTh was associated with a favorable prognosis, very similar to patients with elevated but non-extremely high platelet counts. Patients with high platelets at the time of diagnosis of primary or secondary myelofibrosis appear to have favorable overall survival (OS) and a favorable bleeding risk over time. We did not observe a difference in thrombotic risk based on specific platelet categories, suggesting that neither high nor low platelet counts affect thrombotic risk among MPN patients in the overt myelofibrotic stage. It should be noted that post-ET SMF patients may have the most favorable survival among myelofibrosis subsets [8,58], which may underline the beneficial effects of ExTh on survival observed in the current study. Additionally, a more favorable prognosis may influence the relationship between older age and ExTh at the time of SMF diagnosis. A similar pattern could be observed for palpable spleen size, which also tends to be less enlarged in post-ET SMF patients compared to other subsets. This raises the issue of optimal timing and clinical thresholds for performing bone marrow biopsies, particularly in patients classified as having more indolent MPN subtypes, such as ET. Multiple biopsies are often required to establish a diagnosis of post-ET SMF or post-PV SMF, primarily due to the requirement of at least grade II bone marrow fibrosis. Since there are no standardized recommendations, many patients with prior diagnoses of ET, PV, or pre-PMF go undetected when they “truly” progress, as the decision to perform a repeat bone marrow biopsy is typically based on individual physician discretion. Consequently, specialized care for MPN patients becomes critical, and treating physicians should remain attentive to clinical features and symptoms that may signal disease progression. Currently, more clinical evidence is needed to guide informed decision-making.

The main limitations of the current study are its retrospective design and the loss of statistical power due to the small number of patients in specific subgroups. No causal relationships could be investigated because of the study design’s inherent limitations. Additionally, a number of unmeasured variables may have influenced the reported observations. No information was available on secondary von Willebrand status or the number of bone marrow biopsies performed prior to the diagnosis of overt myelofibrosis. Additionally, the majority of patients lacked data on karyotype, molecular mutations beyond driver mutations, or the allele burden of driver mutations, limiting insights into the relationship of these factors with ExTh. Consequently, we were unable to thoroughly investigate this issue. All patients were of Caucasian race, which limits the generalizability of our findings to other racial and national contexts. Nevertheless, this study is the first to evaluate this rare phenomenon using a multi-institutional registry approach and to report its clinical and prognostic associations.

## 5. Conclusions

ExTh is rare among myelofibrosis patients at the time of diagnosis and is associated with the post-ET etiology of myelofibrosis, older age, smaller spleen size, and the presence of arterial hypertension. Aside from higher aspirin use, ExTh showed no association with the utilization of other specific therapies. ExTh was associated with favorable survival and a lower bleeding risk but had no significant association with thrombotic risk.

## Figures and Tables

**Table 1:** Patients’ characteristics stratified according to the presence of extreme thrombocytosis.

	Platelets > 1000 × 10^9^/L	Platelets ≤ 1000 × 10^9^/L	*p* Value
**Nm. of patients**	10 (5.8%)	162 (94.2%)	-
**Age** (years)	72 IQR (70.25–80)	68 IQR (61–76)	0.010 *
**Male sex**	6/10 (60%)	95/162 (58.6%)	1.000
**Etiology of myelofibrosis** PMF Post-PV SMF Post-ET SMF	5/10 (50%) 0/10 (0%) 5/10 (50%)	96/162 (59.3%) 37/162 (22.8%) 29/162 (17.9%)	0.025 *
**BM fibrosis** Grade II Grade III	8/10 (80%) 2/10 (20%)	104/162 (64.2%) 58/162 (35.8%)	0.497
***JAK2*** **mutated**	7/9 (77.8%)	113/153 (73.9%)	1.000
***CALR*** **mutated**	0/7 (0%)	11/128 (8.6%)	1.000
***MPL*** **mutated**	1/7 (14.3%)	3/126 (2.4%)	0.197
**Constitutional symptoms**	3/10 (30%)	86/162 (53.1%)	0.200
**Transfusion dependency**	5/10 (50%)	46/162 (28.4%)	0.164
**Massive splenomegaly**	1/10 (10%)	48/150 (32%)	0.286
**Spleen size under left costal margin** (cm)	0.5 IQR (0–1.75)	5 IQR (1–10)	0.015 *
**WBC** (×10^9^/L)	15.1 IQR (11.18–24.65)	10.6 IQR (6.03–19.08)	0.119
**Circulatory blasts ≥1%**	3/10 (30%)	73/162 (45.1%)	0.515
**ANC** (×10^9^/L)	11.2 IQR (9.45–21.14)	7.2 IQR (3.3–13.46)	0.075
**ALC** (×10^9^/L)	1.6 IQR (1.38–2.53)	1.4 IQR (1–2.15)	0.202
**Abs. mono.** (×10^9^/L)	0.6 IQR (0.48–1.53)	0.4 IQR (0.21–0.81)	0.114
**Abs. basophils** (×10^9^/L)	0.2 IQR (0.1–0.3)	0.1 IQR (0.05–0.3)	0.643
**NLR**	5.8 IQR (4.49–8.31)	4.3 IQR (2.36–8.6)	0.205
**Hemoglobin level** (g/L)	108.5 IQR (80.25–110.75)	102.5 IQR (88.25–123)	0.862
**MCV** (fL)	94.2 IQR (89–96)	88 IQR (82.05–93.15)	0.137
**MCHC** (g/L)	313 IQR (293–315)	318.5 IQR (307–329.25)	0.013 *
**RDW** (%)	19.3 IQR (17.3–25.4)	19.6 IQR (18.25–21.75)	0.912
**Platelets** (×10^9^/L)	1214.5 IQR (1147.25–1504.5)	298.5 IQR (172.5–472.5)	<0.001 *
**MPV** (fL)	9 IQR (8.25–9.83)	9.7 IQR (8.5–10.63)	0.304
**LDH** (U/L)	391 IQR (317.25–837.5)	504.5 IQR (348–742.25)	0.701
**CRP** (mg/L)	6.7 IQR (2.7–9.7)	5.9 IQR (2.3–13.3)	0.763
**Albumin** (g/L)	42.5 IQR (36.75–47)	42 IQR (39–44.33)	0.966
**Uric acid** (mmol/L)	513 IQR (418–594.5)	383.5 IQR (319–467)	0.055
**Ferritin** (mcg/L)	147 IQR (61.5–245.48)	211 IQR (77–488.5)	0.502
**Serum creatinine** (mcmol/L)	112 IQR (96.25–142.75)	84 IQR (71–101.25)	0.007 *
**Charlson comorbidity index**	3 IQR (3–4)	3 IQR (2–5)	0.455
**CV risk factors**	10/10 (100%)	99/145 (68.3%)	0.034 *
**Chronic kidney disease**	2/5 (40%)	19/116 (16.4%)	0.207
**Arterial hypertension**	9/10 (90%)	84/147 (57.1%)	0.049
**Diabetes mellitus**	0/10 (0%)	24/149 (16.1%)	0.361
**Hyperlipoproteinemia**	2/10 (20%)	22/140 (15.7%)	0.662
**Obesity**	0/8 (0%)	6/115 (5.2%)	1.000
**Active smoking**	1/8 (12.5%)	18/120 (15%)	1.000
**History of thrombosis**	1/10 (10%)	28/162 (17.3%)	1.000
**DIPSS (PMF)** Low risk Intermediate-1 risk Intermediate-2 risk High risk	0/5 (0%) 2/5 (40%) 1/5 (20%) 2/5 (40%)	4/96 (4.2%) 29/96 (30.2%) 53/96 (55.2%) 10/96 (10.4%)	0.174
**MYSEC-PM (SMF)** Low risk Intermediate-1 risk Intermediate-2 risk High risk	0/3 (0%) 2/3 (66.7%) 1/3 (33.3%) 0/3 (0%)	8/57 (14%) 20/57 (35.1%) 14/57 (24.6%) 15/57 (26.3%)	0.557

* statistically significant at level *p* < 0.05/Abbreviations: IQR—interquartile range, PMF—primary myelofibrosis, PV—polycythemia vera, SMF—secondary myelofibrosis, ET—essential thrombocythemia, BM—bone marrow, JAK2—Janus kinase 2, CALR—Calreticulin, MPL—thrombopoietin receptor, Massive splenomegaly—palpable spleen ≥10 cm under the left costal margin, WBC—white blood cells, ANC—absolute neutrophil count, ALC—absolute lymphocyte count, Abs.—absolute, NLR—neutrophil to lymphocyte ratio, MCV—mean corpuscular volume, MCHC—mean corpuscular hemoglobin concentration, RDW—red cell distribution width, MPV—mean platelet volume, LDH—lactate dehydrogenase, CRP—C reactive protein, CV—cardiovascular, DIPSS—Dynamic International Prognostic Scoring System, MYSEC-PM—myelofibrosis secondary to PV and ET prognostic model.

## Data Availability

The raw data supporting the conclusions of this article will be made available by the authors upon request.
